# Metabolic Reconstruction Elucidates the Lifestyle of the Last Diplomonadida Common Ancestor

**DOI:** 10.1128/mSystems.00774-20

**Published:** 2020-12-22

**Authors:** Alejandro Jiménez-González, Jan O. Andersson

**Affiliations:** a Uppsala Biomedicine Centre, Department of Cell and Molecular Biology, Molecular Evolution program, Uppsala University, Uppsala, Sweden; University of California, San Diego

**Keywords:** ancestral reconstruction, lateral gene transfer, metabolism, parasites, protists

## Abstract

The identification of ancestral traits is essential to understanding the evolution of any group. In the case of parasitic groups, this helps us understand the adaptation to this lifestyle and a particular host. Most diplomonads are parasites, but there are free-living members of the group nested among the host-associated diplomonads. Furthermore, most of the close relatives within Fornicata are free-living organisms. This leaves the lifestyle of the ancestor unclear. Here, we present metabolic maps of four different diplomonad species. We identified 853 metabolic reactions and 147 pathways present in at least one of the analyzed diplomonads. Our study suggests that diplomonads represent a metabolically diverse group in which differences correlate with different environments (e.g., the detoxification of arsenic). Using a parsimonious analysis, we also provide a description of the putative metabolism of the last Diplomonadida common ancestor. Our results show that the acquisition and loss of reactions have shaped metabolism since this common ancestor. There is a net loss of reaction in all branches leading to parasitic diplomonads, suggesting an ongoing reduction in the metabolic capacity. Important traits present in host-associated diplomonads (e.g., virulence factors and the synthesis of UDP-*N-*acetyl-d-galactosamine) are shared with free-living relatives. The last Diplomonadida common ancestor most likely already had acquired important enzymes for the salvage of nucleotides and had a reduced capacity to synthesize nucleotides, lipids, and amino acids *de novo*, suggesting that it was an obligate host-associated organism.

**IMPORTANCE** Diplomonads are a group of microbial eukaryotes found in oxygen-poor environments. There are both parasitic (e.g., Giardia intestinalis) and free-living (e.g., *Trepomonas*) members in the group. Diplomonads are well known for their anaerobic metabolism, which has been studied for many years. Here, we reconstructed whole metabolic networks of four extant diplomonad species as well as their ancestors, using a bioinformatics approach. We show that the metabolism within the group is under constant change throughout evolutionary time, in response to the environments that the different lineages explore. Both gene losses and gains are responsible for the adaptation processes. Interestingly, it appears that the last Diplomonadida common ancestor had a metabolism that is more similar to extant parasitic than free-living diplomonads. This suggests that the host-associated lifestyle of parasitic diplomonads, such as the human parasite *G. intestinalis*, is an old evolutionary adaptation.

## Abstract (precis)

Diplomonads are a group of microbial eukaryotes found in oxygen-poor environments. There are both parasitic (e.g., Giardia intestinalis) and free-living (e.g., *Trepomonas*) members in the group.

## INTRODUCTION

The identification of the metabolic capacities of any species or group is an important task to understand the adaptation to the environment. For example, it could help to elucidate the interactions with other species *in vivo* and to identify the growth requirement for those organisms that fail to grow in axenic cultures (1, 2). The comparison of the metabolism of different species provides essential data to predict the metabolism of their common ancestor (3). The description of the ancestral state of a group helps to understand how the different lifestyles evolved within the group.

Diplomonads are an example of a group that is suitable to study lifestyle transitions because they contain both host-associated and free-living species (4). They are flagellated protists found in low-oxygen environments and classified within the group Fornicata (Metamonada) (5). Giardia intestinalis, the causative agent of giardiasis in humans, is the most widely known diplomonad. This organism has been used as a model to understand the evolution of parasitism and the reduction of the mitochondria (6–11). However, members of diplomonads can also colonize other mammals as well as fish, amphibians, and birds (4). Studies of the fish parasite *Spironucleus salmonicida* have deepened the understanding of parasitism in diplomonads (6, 12, 13). *Trepomonas* sp. strain PC1 has been described as a secondary free-living organism because its ancestor escaped a parasitic lifestyle thanks to the acquisition, from bacteria, of many genes associated with its free-living lifestyle (14). Recently, new genomes and transcriptomes have been published from close relatives of diplomonads (6, 15). Interestingly, the closest relatives of diplomonads among these are free-living (6, 16), raising the question of whether the last Diplomonadida common ancestor was already a parasite or if the transition to parasitism occurred multiple times within the group.

A comparison of the metabolic capacities of extant diplomonads could shed light on this question. The metabolism of various diplomonads has indeed been studied, both with experimental and bioinformatic approaches (12, 14, 17–21). Here, we present a systematic comparative reanalysis of the metabolic capacities of four diplomonad species. We show that traits associated with a host-associated lifestyle were present already in the last Diplomonadida common ancestor.

## RESULTS

**FIG 1:**
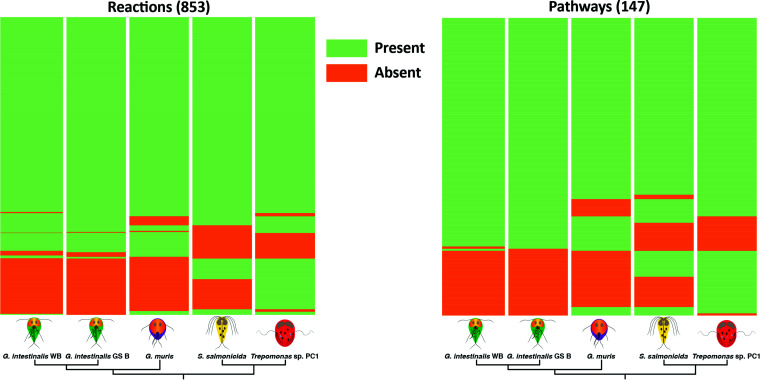
Heatmaps of reactions (left) and pathways (right). Reactions and pathways present and absent in a diplomonad are in green and orange, respectively.

**FIG 2:**
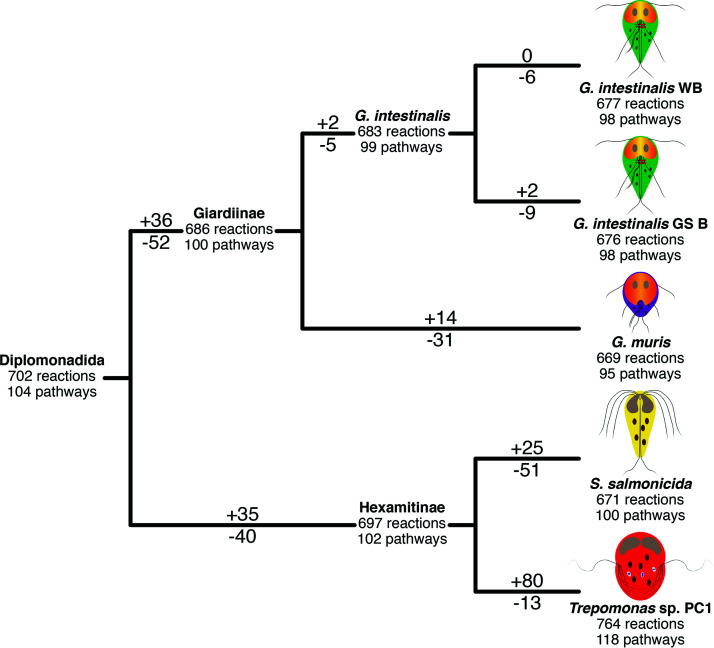
Total reactions and pathways identified in the studied diplomonads and the different ancestors. Positive and negative numbers indicate reactions gained and lost at that branch.

We manually curated the annotations of genes coding for metabolic reactions in four genomes and one transcriptome representing four diplomonad species using a number of prediction tools and databases (see Materials and Methods for details). In total, we identified 853 reactions (see Table S1 in the supplemental material) and 147 pathways present in at least one of the analyzed diplomonads. Among these, 559 reactions (66%) and 82 pathways (56%) were common to all the analyzed diplomonads, while 101 reactions (12%) and 22 pathways (15%) were unique to one diplomonad (Fig. 1). *Trepomonas* sp. strain PC1 showed the most complex metabolism, with 764 reactions and 118 pathways, while *G. muris* showed the most reduced metabolism, with 669 reactions and 95 pathways (Fig. 2).

10.1128/mSystems.00774-20.8 TABLE S1 Classifications of all identified reactions. Reactions are identified by the BioCyc ID (first column) and, when possible, by the EC number (second column). Reactions present in the different diplomonad species are marked with an X. Reactions whose BLASTp search retrieved *K. bialata* homologs are also marked with an X. Reactions for which the interpretation was modified based on previous studies are marked by an asterisk. Download Table S1, XLSX file, 0.07 MB. Copyright © 2020 Jiménez-González and Andersson. 2020 Jiménez-González and Andersson. This content is distributed under the terms of the Creative Commons Attribution 4.0 International license.

We constructed clusters of genes based on their functional annotation. The evolutionary origin of the genes in the clusters was predicted based on the identity of the most similar sequences (see Materials and Methods for details and Table S1). The putative metabolic capacities of the last Diplomonadida common ancestor and the Giardiinae and Hexamitinae ancestors were reconstructed using this approach (Fig. 2 and Fig. S1 to S6).

10.1128/mSystems.00774-20.1 FIG S1 Metabolic map of the last Diplomonadida common ancestor. Reactions that clustered with *K. bialata* and were classified as vertically inherited are in red. Reactions that did not cluster with *K. bialata* and were classified as vertically inherited are in orange. Reactions that clustered with *K. bialata* and were classified as LGT candidates are in green. Reactions that did not cluster with *K. bialata* and were classified as LGT candidates are in pink. Spontaneous reactions are in blue. Gaps in pathways are in grey. Download FIG S1, PDF file, 0.3 MB. Copyright © 2020 Jiménez-González and Andersson. 2020 Jiménez-González and Andersson. This content is distributed under the terms of the Creative Commons Attribution 4.0 International license.

### Last Diplomonadida common ancestor.

The last Diplomonadida common ancestor encoded 702 of the 853 reactions and 104 of the 147 pathways annotated in the extant species (Fig. 2). The predicted overall metabolism was, as expected, similar to that of extant diplomonads (Fig. S1). For example, the last Diplomonadida common ancestor appeared to have produced pyruvate via glycolysis. Pyruvate was converted to acetyl-coenzyme A (CoA) via pyruvate:ferredoxin oxidoreductase (Fig. S1). This ancestor could produce pyruvate either via pyruvate kinase or via the most efficient pyrophosphate-dependent pyruvate phosphate dikinase enzyme, similar to extant *G. intestinalis* and *Trepomonas* sp. strain PC1. The Diplomonadida ancestor could interconvert phosphoenolpyruvate and oxaloacetate via phosphoenolpyruvate carboxykinase, which allowed it to adjust its metabolism depending on the environmental conditions, similar to Giardiinae species (Fig. S1). The pentose phosphate pathway appears to be partial and use ribulose 5-phosphate or erythrose 4-phosphate and xylulose 5-phosphate to produce glyceraldehyde 3-phosphate. This ancestor could synthesize UDP-*N-*acetyl-d-galactosamine, a compound that is essential for building the cyst wall, together with three cyst wall proteins in *Giardia* species and probably *S. salmonicida* (12, 22). The capacity for synthesizing UDP*-N-*acetyl-d-galactosamine was already present in the common ancestor of diplomonads and *Kipferlia bialata*, suggesting that the Diplomonadida ancestor had the metabolic capacity to form cysts (Fig. S1).

Thirteen proteins have been suggested to be virulence factors in *G. intestinalis*. Most of these enzymes are proteases that disrupt the epithelial cells and the intestinal biofilm of the host (23). Our analysis suggested that most of these proteases were already present in the common ancestor of *K. bialata* and diplomonads (Fig. S1). Only two of the potential virulence factors were not shared with *K. bialata*: uridine phosphorylase and serine peptidase. However, both proteins were classified as vertically inherited candidates and probably were lost in *K. bialata*. These observations suggest that the candidate virulence factors evolved in a free-living ancestor of diplomonad parasites, indicating that they are not parasite-specific inventions.

Members of Metamonada that are host associated, such as *G. intestinalis*, *S. salmonicida*, Trichomonas vaginalis, and *Monocercomonoides exilis*, have been shown to have a metabolism that is dependent on the supply of metabolites from the environment within the host (12, 24–26). Our analyses indeed suggested that the Diplomonadida ancestor had a limited capacity to synthesize lipids, amino acids, and nucleotides *de novo* (Fig. S1), in agreement with a host-associated lifestyle.

Several transporters of lipids were present, and only pathways for lipid modification were identified, suggesting that it depended on external sources (Fig. S1). The ancestor most likely could utilize arginine, tryptophan, and serine available in the environment as sources of energy. Whereas the arginine dihydrolase pathway is widespread in Metamonada, the capacity for degradation of tryptophan and serine was acquired from bacteria in the lineage leading to the Diplomonadida ancestor.

**FIG 3:**
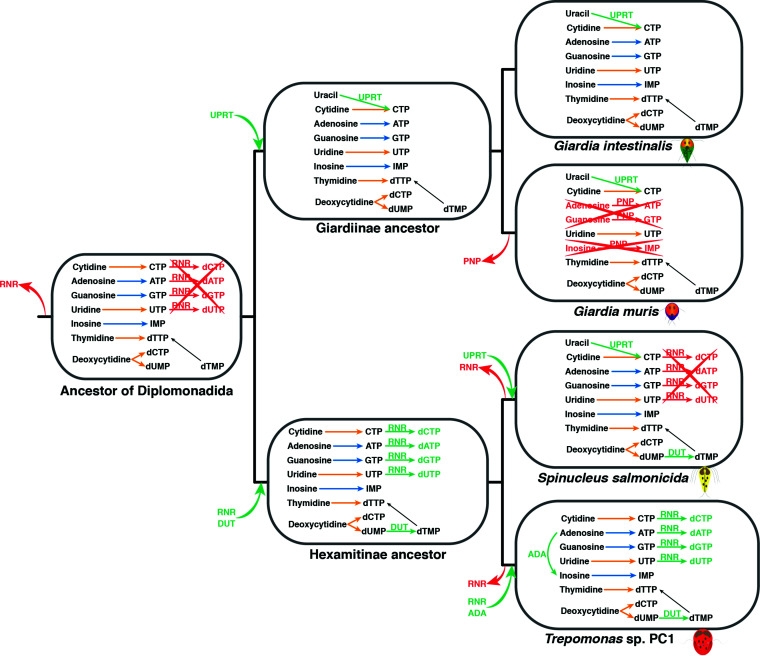
Evolution of the synthesis of nucleotides and nucleosides. Key reactions vertically inherited are in black. Pathways and reactions that clustered with *K. bialata* and were classified as LGT candidates are in orange. Pathways and reactions that did not cluster with *K. bialata* and were classified as LGT candidates are in blue. Pathways and reactions classified as LGT after the last Diplomonadida common ancestor are in green. Pathways and reactions lost are in red. RNR, anaerobic ribonucleoside-triphosphate reductase; UPRT, uracil phosphoribosyltransferase; PNP, purine nucleoside phosphorylase; DUT, deoxyuridine 5′-triphosphate nucleotidohydrolase; ADA, adenosine deaminase.

Similarly, the Diplomonadida ancestor had several pathways for the salvage and degradation of nucleotides and nucleosides and was likely dependent on the salvage pathways to ascertain the availability of nucleotides and deoxynucleotides (Fig. 3 and Fig. S1). All nucleotide salvage pathways appear to have been acquired since the last eukaryotic ancestor. Our analyses classified all key enzymes related to salvage and degradation of purines as lateral gene transfer (LGT) candidates acquired after the divergence from *K. bialata* (Fig. 3 and Fig. S1), whereas the key enzymes for the salvage and degradation of pyrimidines shared an origin with *K. bialata*. This suggests that the Diplomonadida ancestor could salvage nucleosides and convert them into all needed nucleotides (Fig. 3). The enzyme ribonucleotide reductase synthesizes deoxynucleotides from nucleotides. All organisms, except a few parasites, encode this enzyme (27). It was previously shown that anaerobic ribonucleoside-triphosphate reductase is present in some parasitic diplomonads but absent from *G. intestinalis* and *S. salmonicida* (9, 14). Our analysis indicated that the Diplomonadida ancestor lacked the enzyme. A phylogenetic analysis suggested that a bacterial anaerobic ribonucleoside-triphosphate reductase was acquired before the split with *K. bialata* and was subsequently lost in the lineage leading to the last Diplomonadida common ancestor (Fig. 3 and Fig. S7). The enzyme was regained in the Hexamitinae ancestor, lost in several lineages, and gained a third time via LGT by *Trepomonas* sp. strain PC1 (14). Thus, it appears that the last Diplomonadida common ancestor was dependent on a source of deoxynucleotides for the synthesis of DNA, similar to *Giardia* and *S. salmonicida*.

### Giardiinae ancestor, Giardia intestinalis, and *Giardia muris*.

All members of Giardiinae are parasites, and a reduction of the metabolic capacity could be expected within the group. Fifty-two reactions were indeed lost in the lineage from the last Diplomonadida ancestor to the Giardiinae ancestor (Fig. 2 and Fig. S2A). However, 36 reactions were also gained, suggesting an evolutionary flexibility of the metabolic capacities (Fig. 2 and Fig. S2B). The nucleotide metabolism was affected by the loss of the last step for the degradation of adenosine, guanosine, and inosine-5-phosphate. However, uracil phosphoribosyltransferase was gained in the lineage leading to this ancestor, independent of the gain event in *S. salmonicida* (Fig. 3). This acquisition expanded the possibilities for nucleotide salvage. The energy metabolism was also affected because the ability to degrade galactose and triacylglycerols was lost (Fig. S2A). Our analyses suggested that the Giardiinae ancestor acquired the oxidative branch of the pentose phosphate pathway (Fig. S2B). This pathway synthesizes ribulose 5-phosphate and NADH from glucose 6-phosphate. The NADH is used in different reactions, including oxygen detoxification. The ribulose 5-phosphate is used to synthesize glyceraldehyde 3-phosphate through the partial pentose phosphate pathway already present in the last Diplomonadida common ancestor. The glyceraldehyde 3-phosphate can be used to synthesize pyruvate in the last steps of glycolysis. Interestingly, our analyses suggested that the Giardiinae ancestor lost the transporters of glyceraldehyde 3-phosphate. This loss could be related to the acquisition of this oxidative branch. This ancestor could take glucose from glycogen, a trait shared with *Trepomonas* sp. strain PC1. However, the genes in the two diplomonad lineages have independent LGT origins.

10.1128/mSystems.00774-20.2 FIG S2 Reactions lost in the Giardiinae ancestor (A) and metabolic map and LGT candidates in the Giardiinae ancestor (B). Reactions present in the last Diplomonadida common ancestor and lost in the Giardiinae ancestor are in orange. Reactions classified as LGT candidates are in green. Gaps in pathways are in grey. Download FIG S2, PDF file, 0.5 MB. Copyright © 2020 Jiménez-González and Andersson. 2020 Jiménez-González and Andersson. This content is distributed under the terms of the Creative Commons Attribution 4.0 International license.

Only two reactions were classified as LGT candidates in the *G. intestinalis* ancestor since the Giardiinae ancestor, MsrA and flavohemoprotein, both of which have been reported before (28) (Fig. 2 and Table S2). Our analyses identified that the *G. intestinalis* ancestor lost five reactions (Fig. 2 and Table S2). This ancestor lacked the protein tryptophanase, which degrades tryptophan to pyruvate, indole, and ammonium. While pyruvate is used to produce energy, indole could interact with the microbiota of the host, interfering with the quorum sensing (29). Our analyses also detected that the *G. intestinalis* ancestor lacked an amino acid transporter. We could not identify the nature of this transporter, but its loss could be connected with the loss of the tryptophanase activity.

10.1128/mSystems.00774-20.9 TABLE S2 Gains and losses in *G. intestinalis* ancestor, *G. intestinalis* WB, and *G.intestinalis* GS B. Download Table S2, PDF file, 0.2 MB. Copyright © 2020 Jiménez-González and Andersson. 2020 Jiménez-González and Andersson. This content is distributed under the terms of the Creative Commons Attribution 4.0 International license.

Our analysis showed that *G. intestinalis* WB and *G. intestinalis* GS B are not metabolically identical (Fig. 1). We identified six reactions lost in *G. intestinalis* WB and nine lost in *G. intestinalis* GS B (Fig. 2 and Table S2). At the same time, we classified two reactions as LGT candidates in *G. intestinalis* GS B since the *G. intestinalis* common ancestor and none in *G. intestinalis* WB (Fig. 2 and Table S2). These differences made these two isolates metabolically distinct. Giardia intestinalis WB lost the ability to degrade arginine to l-ornithine and urea, making the deimination of arginine to l-citrulline the only option to degrade this amino acid. Giardia intestinalis GS B shared the ability to degrade arginine through two different pathways with *G. muris* (30). On the other hand, *G. intestinalis* GS B lost the synthesis of glycine from glyoxylate.

Another significant difference between the two *G. intestinalis* isolates is the absence of the protein quorum-quenching *N*-acyl-homoserine lactonase in *G. intestinalis* WB (Table S2). This protein interferes with the quorum sensing of different bacteria and was previously reported as laterally acquired from bacteria in the Giardiinae ancestor (30).

*Giardia muris* showed the most reduced metabolism among the analyzed diplomonads (Fig. 2). *Giardia muris* lacked most of the pathways related to the salvage and degradation of nucleosides and nucleotides (Fig. S3A). Only the salvage and degradation of pyrimidines are retained (Fig. 3). The absence of those pathways suggested that *G. muris* is highly dependent on a supply of nucleosides and nucleotides, especially purines, from the environment within the host. On the other hand, *G. muris* can synthesize coenzyme A *de novo* (30). Our analysis suggested that the genes responsible for the synthesis of coenzyme A in *G. muris* were acquired independently in the lineages leading to *G. muris* and *S. salmonicida* (Fig. S3B and Table S1)

10.1128/mSystems.00774-20.3 FIG S3 Reactions lost in *G. muris* (A) and metabolic map and LGT candidates in *G. muris* (B). Reactions present in the Giardiinae ancestor and lost in *G. muris* are in orange. Reactions classified as LGT candidates are in green. Gaps in pathways are in grey. Download FIG S3, PDF file, 0.5 MB. Copyright © 2020 Jiménez-González and Andersson. 2020 Jiménez-González and Andersson. This content is distributed under the terms of the Creative Commons Attribution 4.0 International license.

### Hexamitinae ancestor, *Spironucleus salmonicida*, and *Trepomonas* sp. strain PC1.

The lineage from the last Diplomonadida common ancestor to the last Hexamitinae common ancestor has experienced 35 gains and 40 losses of reactions, numbers that are similar to those of the Giardiinae lineage (Fig. 2). Among the losses are the ability to interconvert phosphoenolpyruvate and oxaloacetate via phosphoenolpyruvate carboxykinase and the degradation of arginine through the enzyme arginase (Fig. S4A). This ancestor also lost the synthesis of farnesyl and geranyl diphosphate from isopentenyl pyrophosphate. These losses most likely are related to the absence of the mevalonate pathway, an intermediary pathway for the synthesis of both compounds. In contrast, the capacity for nucleotide metabolism was extended. The enzyme anaerobic ribonucleoside-triphosphate reductase was acquired via LGT (Fig. 3 and Fig. S4B and S7), allowing the organism to synthesize deoxynucleotide triphosphates (dNTPs) from NTPs. Our analysis also suggested that this ancestor acquired the enzyme deoxyuridine 5′-triphosphate nucleotidohydrolase, which converts dUMP into dTMP, which later is converted to dTTP (Fig. 3 and Fig. S4B). Several enzymes classified as LGT candidates are related to the degradation of sugars and proteins (Fig. S4B), suggesting an adaptation to use compounds that this ancestor could find within the host.

10.1128/mSystems.00774-20.4 FIG S4 Reactions lost in the Hexamitinae ancestor (A) and metabolic map and LGT candidates in the Hexamitinae ancestor (B). Reactions present in the last Diplomonadida common ancestor and lost in the Hexamitinae ancestor are in orange. Reactions classified as LGT candidates are in green. Gaps in pathways are in grey. Download FIG S4, PDF file, 0.5 MB. Copyright © 2020 Jiménez-González and Andersson. 2020 Jiménez-González and Andersson. This content is distributed under the terms of the Creative Commons Attribution 4.0 International license.

**FIG 4:**
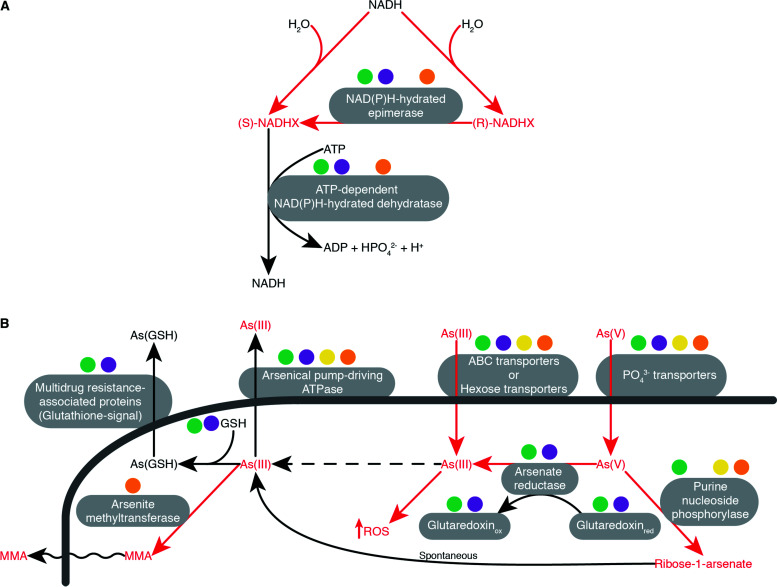
Distribution of proteins across diplomonads for the NADH repair pathway (A) and the arsenic detoxification pathway (B). The presence of proteins and molecules in the studied species are indicated by filled circles: *G. intestinalis* (green), *G. muris* (purple), *S. salmonicida* (yellow), and *Trepomonas* sp. strain PC1 (orange). Toxic compounds and reactions for the cell are in red. NADHX, NADH hydrated; As(V), arsenate; As(III), arsenite; GSH, glutathione; As(GSH), glutathione-chelated-arsenic; MMA, monomethylarsonic acid.

Both analyzed Hexamitinae species showed more pathways than any of the Giardiinae species, although the number of reactions was slightly lower in *S. salmonicida* than *G. intestinalis* (Fig. 2). Notable losses in *S. salmonicida* are the lack of anaerobic ribonucleoside-triphosphate reductase, enzymes for degradation of adenosine, guanosine, and inosine 5-phosphate to urate, and enzymes to repair NADHX (Fig. 4A and Fig. S5A). NADH can be hydrated into NADHX due to the action of some dehydrogenases or spontaneously produced under acidic conditions. NADHX is a toxic compound that inhibits several dehydrogenases (31), and the lack of repair enzymes likely makes *S. salmonicida* sensitive to this compound.

10.1128/mSystems.00774-20.5 FIG S5 Reactions lost in *S. salmonicida* (A) and metabolic map and LGT candidates in *S. salmonicida* (B). Reactions present in the Hexamitinae ancestor and lost in *S. salmonicida* are in orange. Reactions classified as LGT candidates are in green. Gaps in pathways are in grey. Download FIG S5, PDF file, 0.5 MB. Copyright © 2020 Jiménez-González and Andersson. 2020 Jiménez-González and Andersson. This content is distributed under the terms of the Creative Commons Attribution 4.0 International license.

Our analyses found differences in the arsenic detoxification pathway within diplomonads. Arsenic has two biologically relevant oxidation states, arsenite [As(III)] and arsenate [As(V)] (32). Both states are toxic to the cell. While As(III) interacts with thiols, increasing the reactive oxygen species (ROS), As(V) replaces phosphate-producing unstable arsenical compounds (33). For these reasons, all species have mechanisms to avoid the damage of these elements (33, 34). Our analyses suggested that Giardiinae species use glutathione for the detoxification of As(III) and As(V) and *Trepomonas* sp. strain PC1 methylates As(III), making it volatile (Fig. 4B). Curiously, we identified the arsenical pump-driving ATPase as the only direct protein responsible for the detoxification of arsenic in *S. salmonicida* (Fig. 4B). This lack of mechanisms suggested that *S. salmonicida* is very sensitive to stress by arsenic.

Our analyses classified several important proteins as LGT candidates in *S. salmonicida* since the divergence from the lineage leading to *Trepomonas* (Fig. 2 and Table S1). One of them is the protein uracil phosphoribosyltransferase. This protein converts uracil into UMP, making *S. salmonicida* more versatile in the salvage of pyrimidines (Fig. 3 and Fig. S5B). Interestingly, mannose-6-phosphate isomerase was acquired three times independently within diplomonads, making *G. muris*, *Trepomonas* sp. strain PC1, and *S. salmonicida* able to utilize mannose (Table S1). The capacity for melibiose degradation showed a similar pattern. Our analyses suggested that this reaction was acquired independently in *S. salmonicida* and *Trepomonas* sp. strain PC1 (Table S1).

*Trepomonas* sp. strain PC1, the only transcriptome within this study, showed the most complex metabolism (Fig. 2). Although the transcriptome appeared to be fairly complete, the 13 losses need to be considered carefully (Fig. S6A). The 80 gains, on the other hand, should be treated as true gains and not putative contaminations, because the transcriptome data have been carefully curated using the fact that Hexamitinae species utilize an alternative genetic code (14).

One of the most significant metabolic changes in *Trepomonas* sp. strain PC1 was related to the synthesis and degradation of nucleosides and nucleotides (Fig. 3 and Fig. S6B). The acquisition of the protein anaerobic ribonucleoside-triphosphate reductase was a clear advantage for *Trepomonas* sp. strain PC1 (14). This protein uses formate and NTPs for the synthesis of dNTPs (Fig. S6B). Our analysis suggested that genes of *Trepomonas* sp. strain PC1 encoded a formate C-acetyltransferase that converts pyruvate into acetyl-CoA and formate. However, a deeper analysis of the sequence revealed that this protein most likely is a 4-hydroxyphenylacetate decarboxylase (35).

10.1128/mSystems.00774-20.6 FIG S6 Reactions lost in *Trepomonas* sp. strain PC1 (A) and metabolic map and LGT candidates in *Trepomonas* sp. strain PC1 (B). Reactions present in the Hexamitinae ancestor and lost in *Trepomonas* sp. strain PC1 are in orange. Reactions classified as LGT candidates are in green. Gaps in pathways are in grey. Download FIG S6, PDF file, 0.6 MB. Copyright © 2020 Jiménez-González and Andersson. 2020 Jiménez-González and Andersson. This content is distributed under the terms of the Creative Commons Attribution 4.0 International license.

10.1128/mSystems.00774-20.7 FIG S7 Phylogenetic tree of anaerobic ribonucleoside-triphosphate reductase. IQtree maximum-likelihood phylogeny of 520 aligned amino acid residues is shown. Branch support represents ultrafast bootstrap support values followed by SH-aLRT bootstrap support values. Download FIG S7, PDF file, 0.6 MB. Copyright © 2020 Jiménez-González and Andersson. 2020 Jiménez-González and Andersson. This content is distributed under the terms of the Creative Commons Attribution 4.0 International license.

We identified the acquisition of a number of reactions related to nucleotide metabolism and degradation of larger molecules, which most likely played important roles in the development of the secondary free-living lifestyle of *Trepomonas* sp. strain PC1, as previously reported (14). The enzymes adenosine deaminase and dihydropyrimidinase made *Trepomonas* less dependent on the salvage of specific intermediates in the nucleotide metabolism, whereas the acquisitions of α-galactosidase, β-d-glucoside glucohydrolase, β-1,2-mannosidase, glucoamylase, and endoglycosylceramidase made it able to utilize a wide range of carbohydrates putatively available for a free-living bacterivore (Fig. S6B). The acquisitions of the enzymes peptidoglycan dl-endopeptidase, *N*-acetylmuramic-l-alanine amidase, and *N*-acetylmuramic acid 6-phosphate etherase were likely directly related to the ability to digest bacteria (14) (Fig. S6B).

## DISCUSSION

We have combined several metabolic prediction tools and created manually curated metabolic databases of four diplomonads. This approach allows us to minimize the errors due to misannotations and to present the most complete metabolic study in diplomonads to date (Fig. 2 and Fig. S1 to S6). We mapped the gains and losses of functions on the phylogenetic tree and show that all branches leading to parasitic diplomonads show a net loss of reactions, suggesting an ongoing reduction in the metabolic capacity (Fig. 2). Even though this reduction is not very pronounced, it is most likely a consequence of a parasitic or host-associated lifestyle (36).

### Diplomonads, a metabolically diverse group.

Our metabolic analysis shows that diplomonads are a metabolically diverse group of organisms. We identified differences between species that indicate that they are adapted to the environments where they are found. For example, *Spironucleus salmonicida* degrades melibiose into galactose and glucose, a capacity shared with *Trepomonas* sp. strain PC1 (Fig. S5B and S6B). However, our analysis suggested that this represents a convergence. Most likely, both species acquired this capacity via LGT independently. Cases of convergence via LGT have been reported before in diplomonads and other eukaryotes (28, 37). This activity is absent in Giardia intestinalis and *Giardia muris*, indicating either that no successful LGT of genes needed to degrade melibiose has occurred yet in these lineages or that this compound is less frequent in the intestinal tract of their hosts.

Detoxification of arsenic is a second example of the metabolic diversity in diplomonads (Fig. 4B). Giardiinae species can degrade both As(V) and As(III), suggesting that both elements are present at dangerous levels in the intestinal tract of their host. Our analysis shows that this detoxification most likely is dependent on glutathione, a thiol absent in *S. salmonicida* and *Trepomonas* sp. strain PC1. In contrast, Hexamitinae species can only detoxify As(III). As a free-living organism, *Trepomonas* sp. strain PC1 most likely is able to escape if it encounters dangerous levels of As(V). Our analyses showed that *S. salmonicida* also could be sensitive to stress by NADHX. Interestingly, NADHX and As(V) are more abundant in acidic environments (31–34), suggesting that *S. salmonicida* has a preference for neutral or alkaline environments (e.g., pH in the fish blood is between 7.4 and 7.5 [38]).

### The last Diplomonadida common ancestor.

We need to trace the origin of traits associated with parasitism and host association in diplomonads to understand if the last common Diplomonadida ancestor already was a host-associated organism. Previous studies have shown that parasitic diplomonads avoid the immune system of the host via the expression of cysteine-rich proteins (12, 39, 40). These proteins have been studied functionally in *G. intestinalis*, where they are present as a large protein family that functions as variant-specific surface proteins (VSPs) exposed on the surface of the trophozoite (22, 25, 41). A large family of cysteine-rich proteins with a domain structure similar to that of VSPs was described in *S. salmonicida*. Even though the proteins are highly divergent between the two parasitic diplomonads, their structural similarities suggest that they have a common origin (12). The draft genome of *Kipferlia bialata* did not contain any similar cysteine-rich proteins (15), indicating that this protein family represents a parasitic innovation present in the last Diplomonadida ancestor.

We have recently shown that the last Diplomonadida common ancestor was well adapted to low-oxygen environments (28). Even though this characteristic evolved in a free-living lifestyle, being able to survive in low-oxygen environments is a requirement to colonize the intestinal tract of the host. Similarly, there is a set of putative virulence factors identified in *G. intestinalis* (23, 42–44). Here, we show that most of these were present in free-living relatives of diplomonads, and they should be viewed as a preparasitic function and not as a parasitic innovation (45). The formation of cysts is also an important trait in host-associated diplomonads. The pathway to synthesize the cyst wall is shared between diplomonads. Experimental tests have shown that cyst wall proteins from *S. salmonicida* are functional in *G. intestinalis*, suggesting that the ancestor had a similar life cycle (12). The formation of the cyst wall also requires UDP-*N*-acetyl-d-galactosamine (10). Here, we show that the synthesis of this sugar was present already in the last common ancestor of *K. bialata* and diplomonads.

Our analyses show that the last Diplomonadida common ancestor shared important traits with free-living relatives, and several enzymes have been classified as acquired via LGT. However, the metabolic reconstruction suggests that this organism had an overall reduced metabolism (Fig. S1). It likely lacked pathways for the synthesis of essential cellular components, like lipids and most amino acids. Our analyses identified several transporters of both compounds and only pathways for lipid modification. This ancestor also had a reduced nucleotide and nucleoside metabolism and likely lacked the capacity for *de novo* synthesis of dNTPs. Instead, it had acquired the ability to salvage nucleotides and nucleosides. The genes for the enzymes responsible for these reactions were acquired most likely via LGT from bacterial donors. The acquisition via LGT of these reactions is not exclusive of diplomonads. The human parasite Cryptosporidium parvum also acquired several enzymes involved in the salvage of nucleotides and nucleosides from bacteria via LGT (46). Studies of the timing of the eukaryotic diversification show that the last common Diplomondida ancestor probably coexisted with bilateral animals (the most probable host) (47). Taken together, all the present and absent traits in the last Diplomonadida common ancestor strongly suggest that this ancestor was already an obligate host-associated organism, if not already a parasite of some animal.

## MATERIALS AND METHODS

### Sources of data.

Protein sequences from genome data sets of Giardia intestinalis WB, *G. intestinalis* GS B, *G. muris*, S*pironucleus salmonicida*, and *Kipferlia bialata* and the transcriptome data set of *Trepomonas* sp. strain PC1 were downloaded from NCBI (48).

### Identification of metabolic capacities.

The metabolic capacities of each genome and transcriptome were predicted with the GhostKOALA tool (genus_prokaryotes + family_eukaryotes + viruses database) implemented in KEGG (49), EggNOG-mapper (DIAMOND mapping mode) (50), and Pathway-Tool v. 21.5 (default setup) (51). Every genome and transcriptome was manually curated, combining the prediction of these three pieces of software under the Pathway-Tool framework (52). In the case of *G. intestinalis* and *S. salmonicida*, the information contained in GiardiaDB (53) was also added at this step.

Each curated database was improved using the Pathway Hole Filler implemented in Pathway-Tool (54) using the different diplomonad databases as training data sets. When possible, the function for transporters was assigned using Transport Inference Parser (55) implemented in Pathway-Tool and verified with the Conserved Domain Database (56).

### Clustering analysis.

Reactions with at least one protein assigned in one curated database were retrieved from Pathway-Tool. Reactions that were predicted to be present in a database but no enzyme could be assigned were not considered for this analysis (i.e., a gap in a pathway). All proteins from the databases of the different organisms were combined in the same data set when they catalyze the same reaction, creating a reaction data set. Every protein in the reaction data set was used as a query in BLASTp searches against a custom protein sequence database made of the diplomonads used in this analysis, the *K. bialata* (15) proteins, and the UniRef90 database (July 2019) (57). For every query, 500 hits were kept with E values of ≤1e−10. For every query, the first 100 hits were extracted for posterior analyses. We tested different numbers of extracted top hits and found that the first 100 hits gave congruent results with most of the previous phylogenetic analyses performed in studies of diplomonad proteins (28, 30).

A pair of diplomonad sequences were considered to cluster together (i.e., having a common evolutionary origin) if they reciprocally were found among the first 100 hits in the respective BLASTp search. Similarly, a diplomonad sequence was considered to cluster with *K. bialata* if a sequence from that species was among the first 100 hits.

### Classification of the reactions and construction of the last Diplomonadida common ancestor.

Based on the BLASTp searches, we used a parsimonious approach to classify the reactions. A reaction was considered potentially present in the last Diplomonadida common ancestor (ancestral) if it was present in all diplomonad species and they clustered together, the reaction was missing from at least one of the diplomonad lineages but the proteins clustered with *K. bialata*, or, in the absence of clustering with *K. bialata*, the majority of hits were eukaryotic homologs. A reaction was classified as potentially gained in the lineages leading to the Giardiinae, *G. intestinalis*, and/or Hexamitiae ancestors if the proteins from each lineage cluster independently of each other and independently of *K. bialata* and the majority of hits were from prokaryote homologs. Any reaction with a majority of hits from prokaryotes was considered an LGT candidate. Reactions whose evolutionary history has been described previously were manually curated to be consistent (14, 28, 30). Classifications of all reactions are listed in Table S1 in the supplemental material.

The pathways in the different ancestors were predicted under Pathway-Tool v. 21.5 based on the reactions classified to be present in that particular ancestor. The pathways were manually curated and kept or removed based on the number of gaps and taxonomic distribution of the pathway.

### Phylogenetic analysis of anaerobic ribonucleoside-triphosphate reductase.

Nucleotide sequences from the transcriptome data sets of the nondiplomonad Fornicata species *Aduncisulcus paluster*, *Carpediemonas membranifera*, *Chilomastix caulleryi*, *Chilomastix cuspidata*, *Dysnectes brevis*, and *Ergobibamus cyprinoides* (6) were downloaded from the Dryad Digital Repository. tBLASTp searches against these transcriptomes were made using *Trepomonas* sp. strain PC1 and *K. bialata* anaerobic ribonucleoside-triphosphate reductase as a query. The obtained hits were translated into amino acid sequences using EMBOSS Sixpack (58) and evaluated using the Conserved Domain Database. Previously used sequences from *S. barkhanus* and *S. vortens* were included in this analysis (14). The result of these procedures was the creation of a curated Metamonada anaerobic ribonucleoside-triphosphate reductase database.

We performed a phylogenetic analysis by following the approach previously described (28). One sequence from *Trepomonas* sp. strain PC1 and one sequence from *K. bialata* homologs were used as a BLASTp query against the NCBI nr database (October 2019). In this case, the optimal number of hits was 10,000. The number of hits in common between both BLASTp searches was calculated with CD-HIT-2D (59) with the default settings. In this case, the proportion of hits in common was 70%, and both BLASTp searches were merged into a single diversity matrix. This matrix was filtered using CD-HIT (59) by keeping only sequences with <90% sequence identity to another sequence in the data set. This filtered matrix then was merged with homologous proteins from the curated Metamonada anaerobic ribonucleoside-triphosphate reductase database that we had created previously (described above) and aligned using MAFFT v6.603b (60) with the default settings. The resulting alignment was trimmed using BMGE v1.12 (BLOSUM30 with a block size of 2) (61). A preliminary phylogenetic tree was computed using FastTree v2.1.8 SSE3, with OpenMP (62) with default settings, and sequences with a phylogenetic distance of <0.3 were removed in an iterative process to further reduce the size of the matrices until the final matrix was generated.

The final matrix was aligned using MAFFT and trimmed using BMGE, as described above. Maximum likelihood trees were computed using IQtree v. 1.5.3 (63) under the LGX substitution model. Branch supports were assessed using ultrafast bootstrap approximation (UFboot) with 1,000 bootstrap replicates (64) and SH-like approximate likelihood ratio test (SH-aLRT) (65), for which 1,000 replicates were used.
