# Supplementary figures and images for: Metabolic Reconstruction Elucidates the Lifestyle of the Last Diplomonadida Common Ancestor
Source: mSystems. 2020 Dec 22;5(6):e00774-20. doi: 10.1128/mSystems.00774-20 (PMC7762791; doi:10.1128/mSystems.00774-20)

Supplementary Figure 1: Last Diplomonadida common ancestor

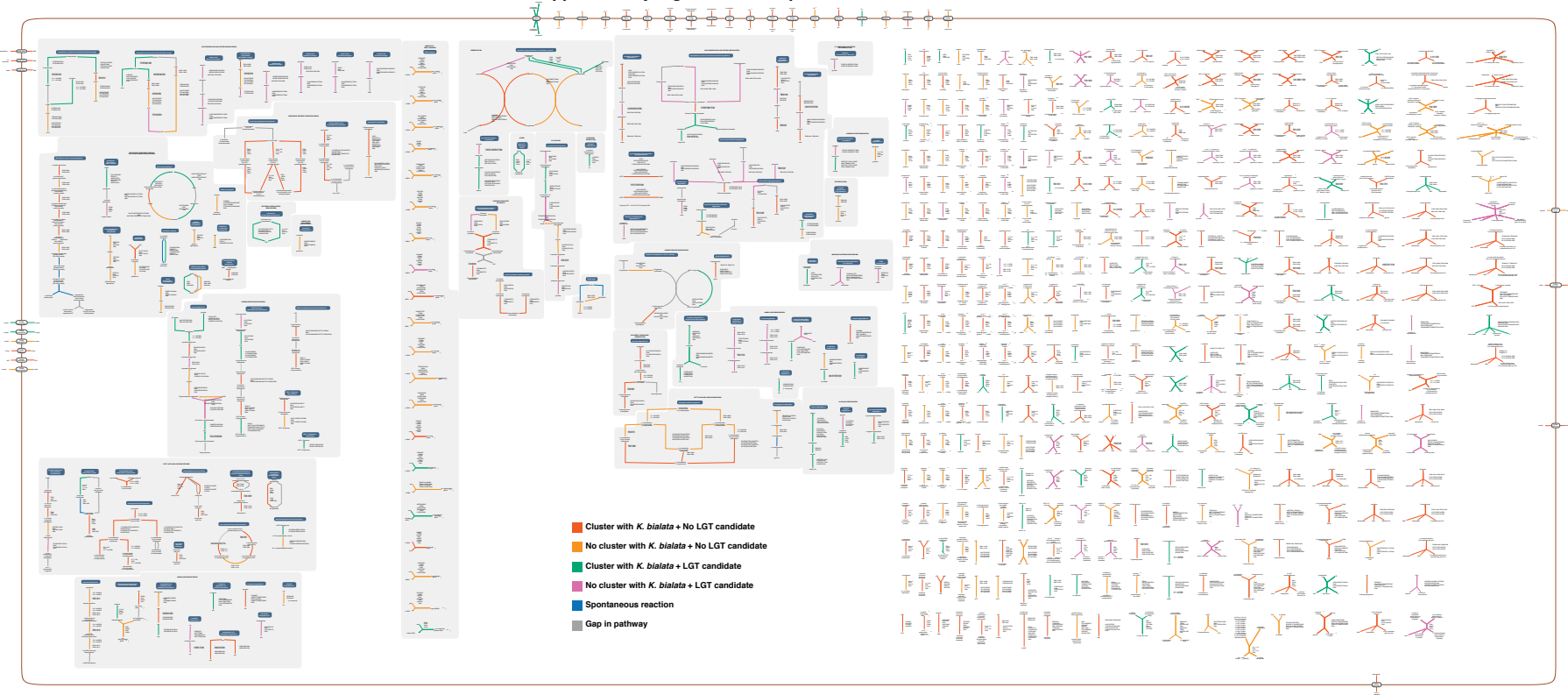

Supplement: FIG S1 [file mSystems.00774-20-sf001.pdf]

Supplementary Figure 2: Reactions lost in *Giardiinae* and LGT candidates

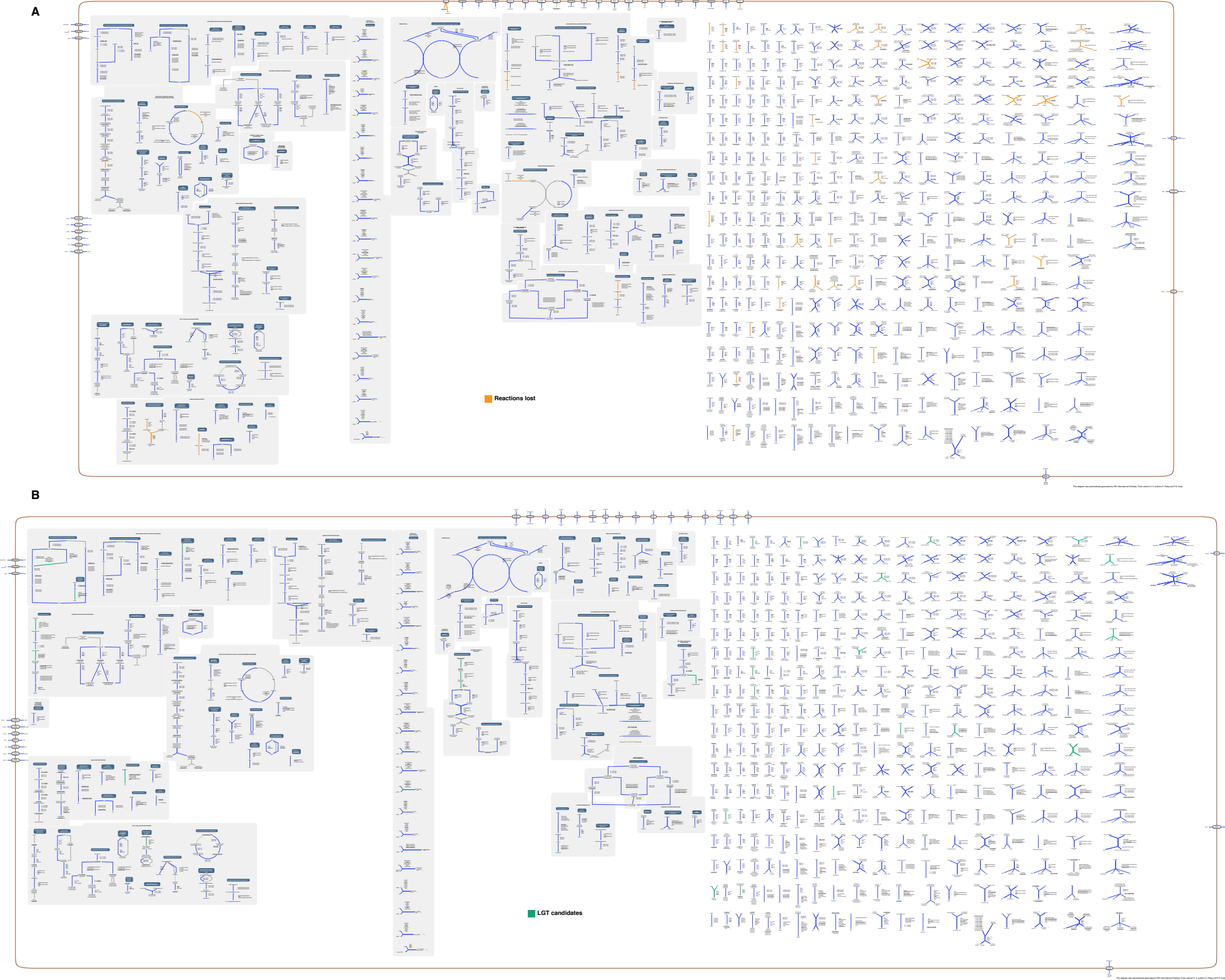

Supplement: FIG S2 [file mSystems.00774-20-sf002.pdf]

Supplementary Figure 3: Reactions lost in *G. muris* and LGT candidates

A

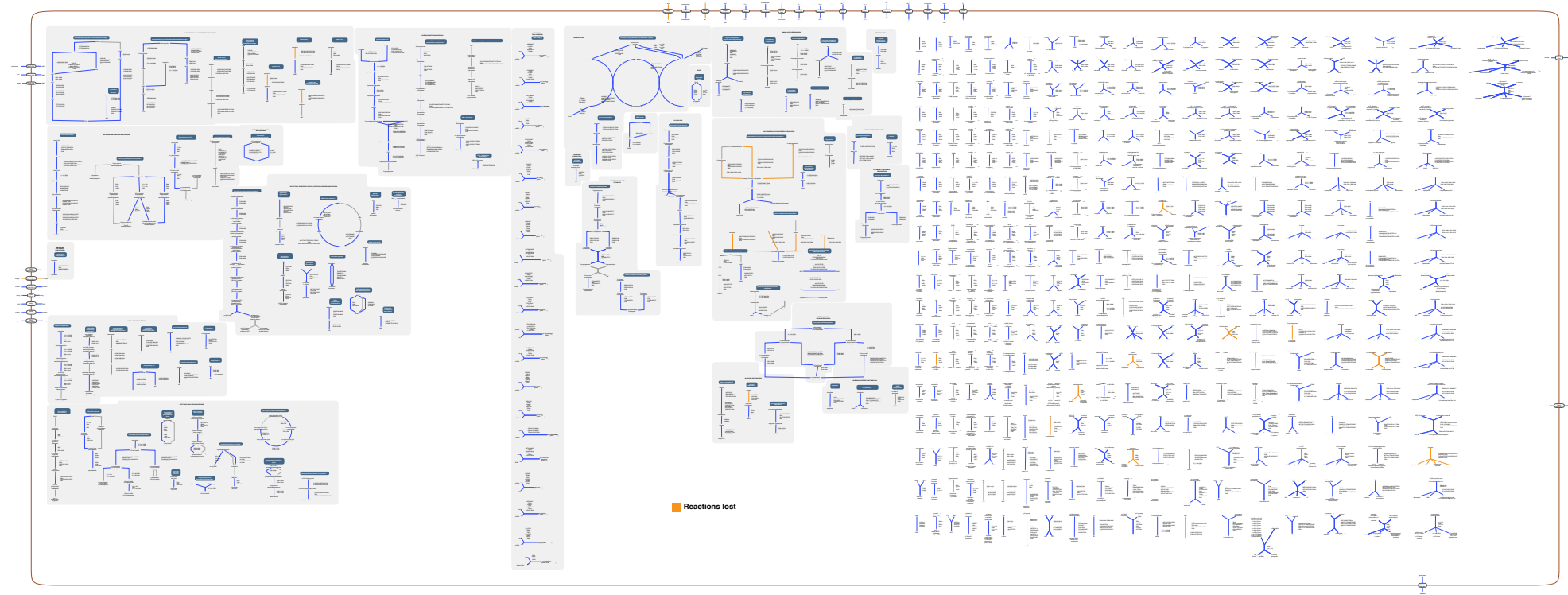

B

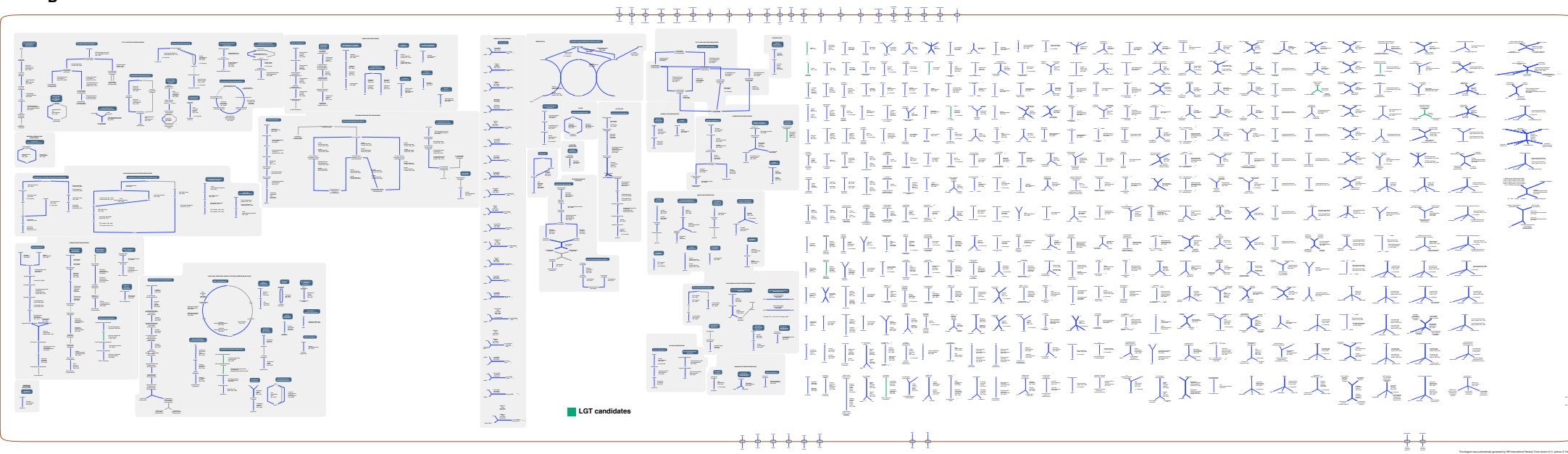

Supplement: FIG S3 [file mSystems.00774-20-sf003.pdf]

Supplementary Figure 4: Reactions lost in Hexamitinae and LGT candidates

A

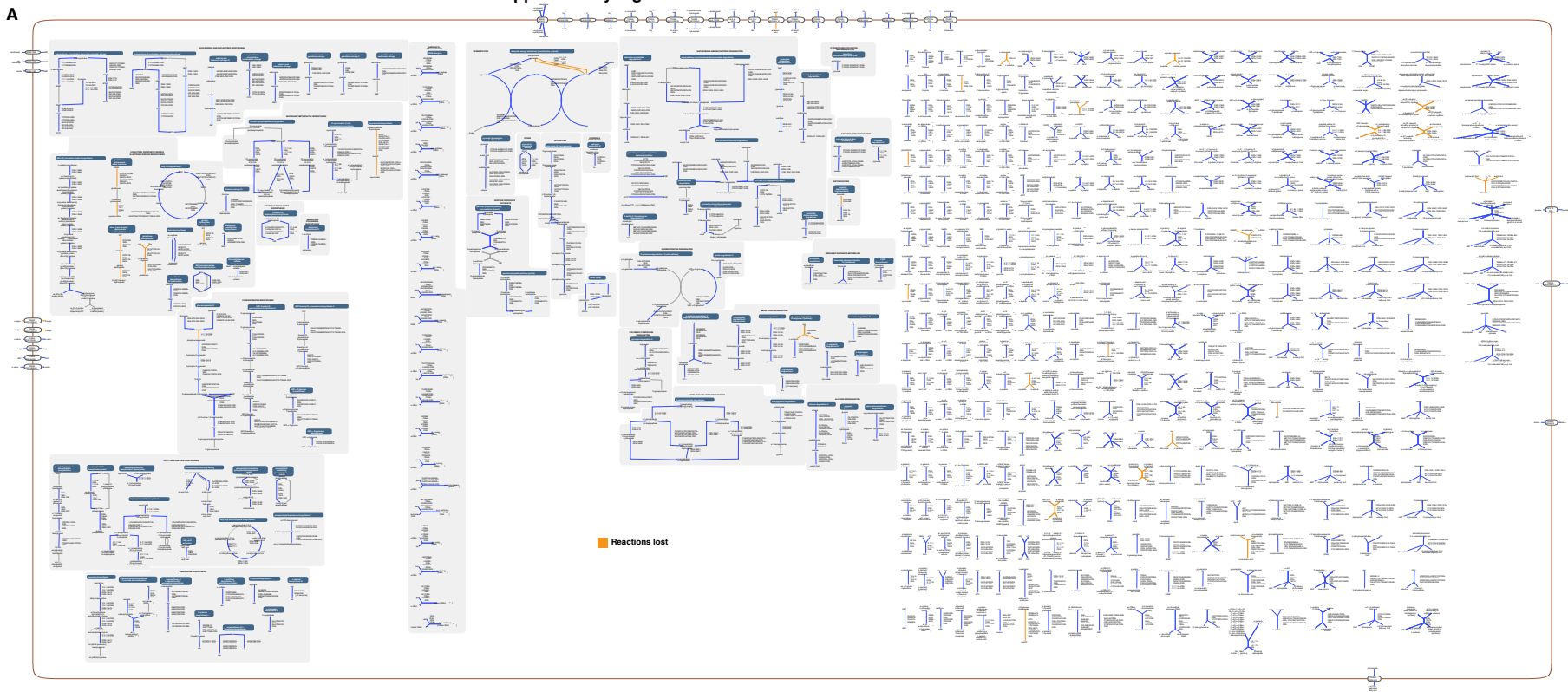

B

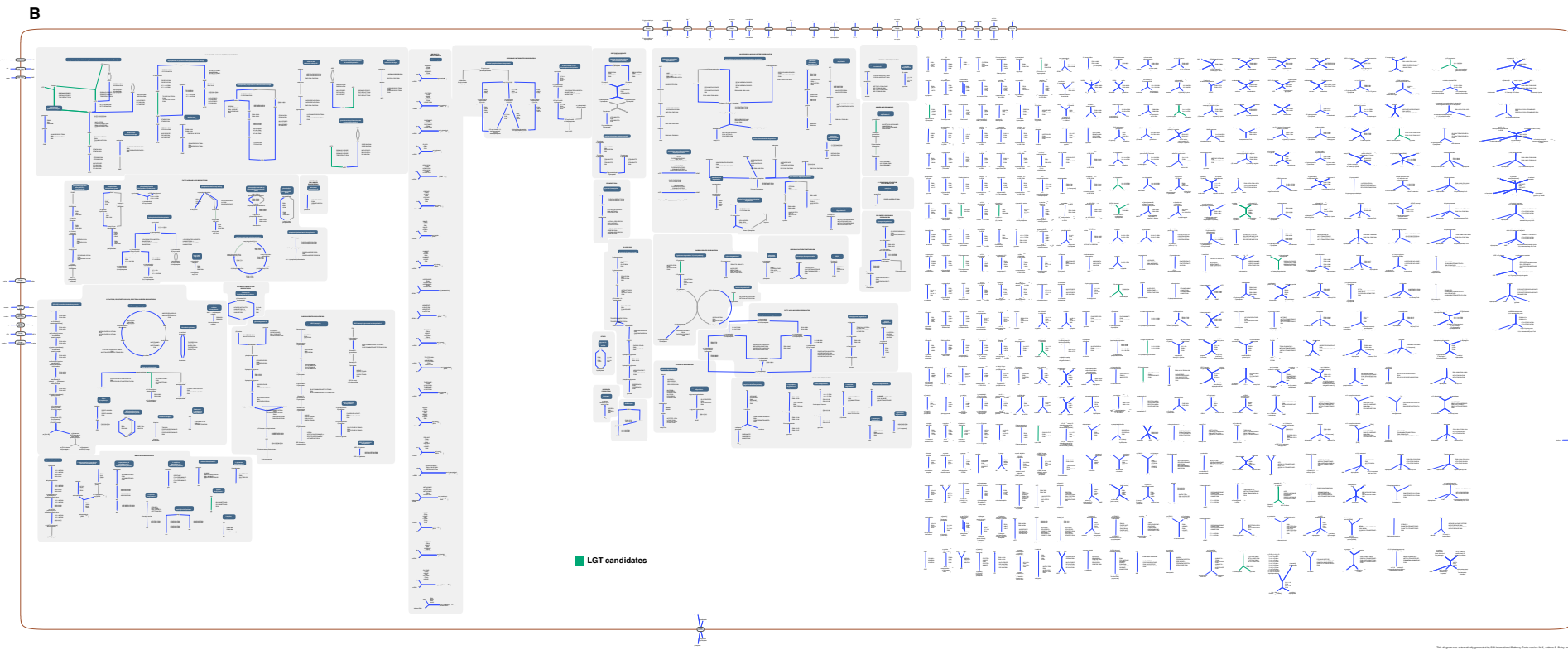

Supplement: FIG S4 [file mSystems.00774-20-sf004.pdf]

Supplementary Figure 5: Reactions lost in *S. salmonicida* and LGT candidates

A

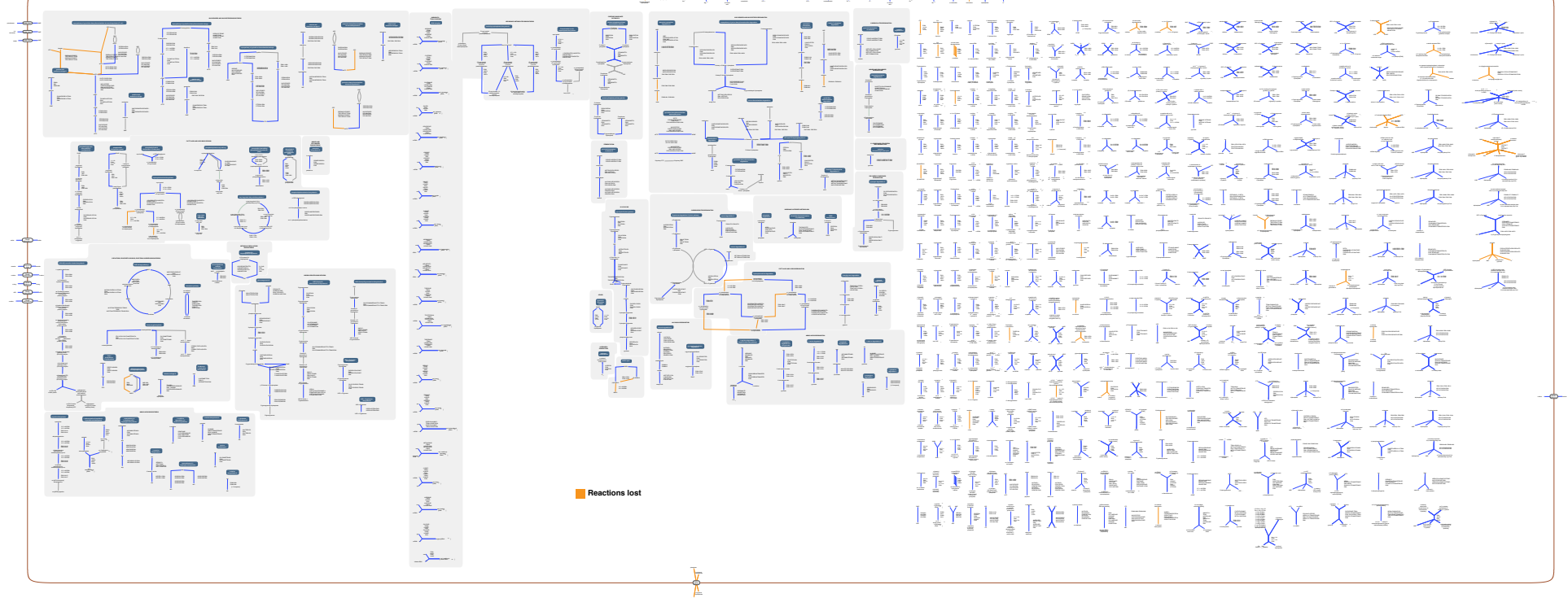

B

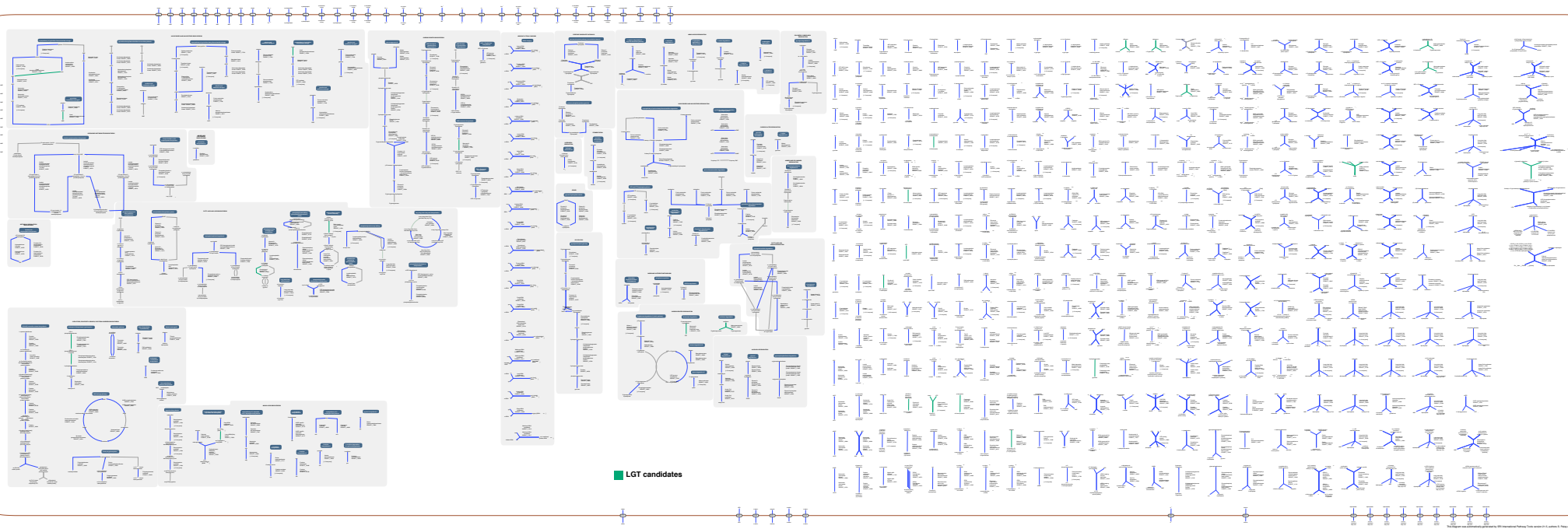

Supplement: FIG S5 [file mSystems.00774-20-sf005.pdf]

Supplementary Figure 6: Reactions lost in *Trepomonas* sp. PC1 and LGT candidates

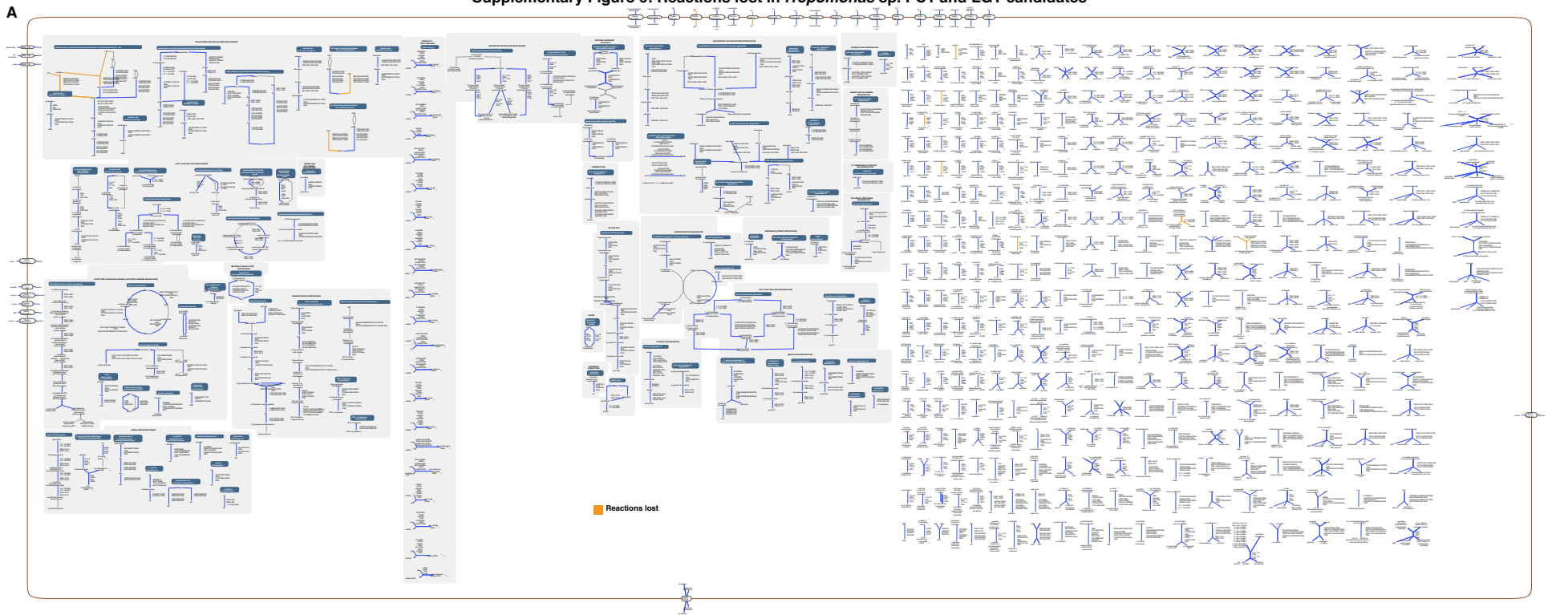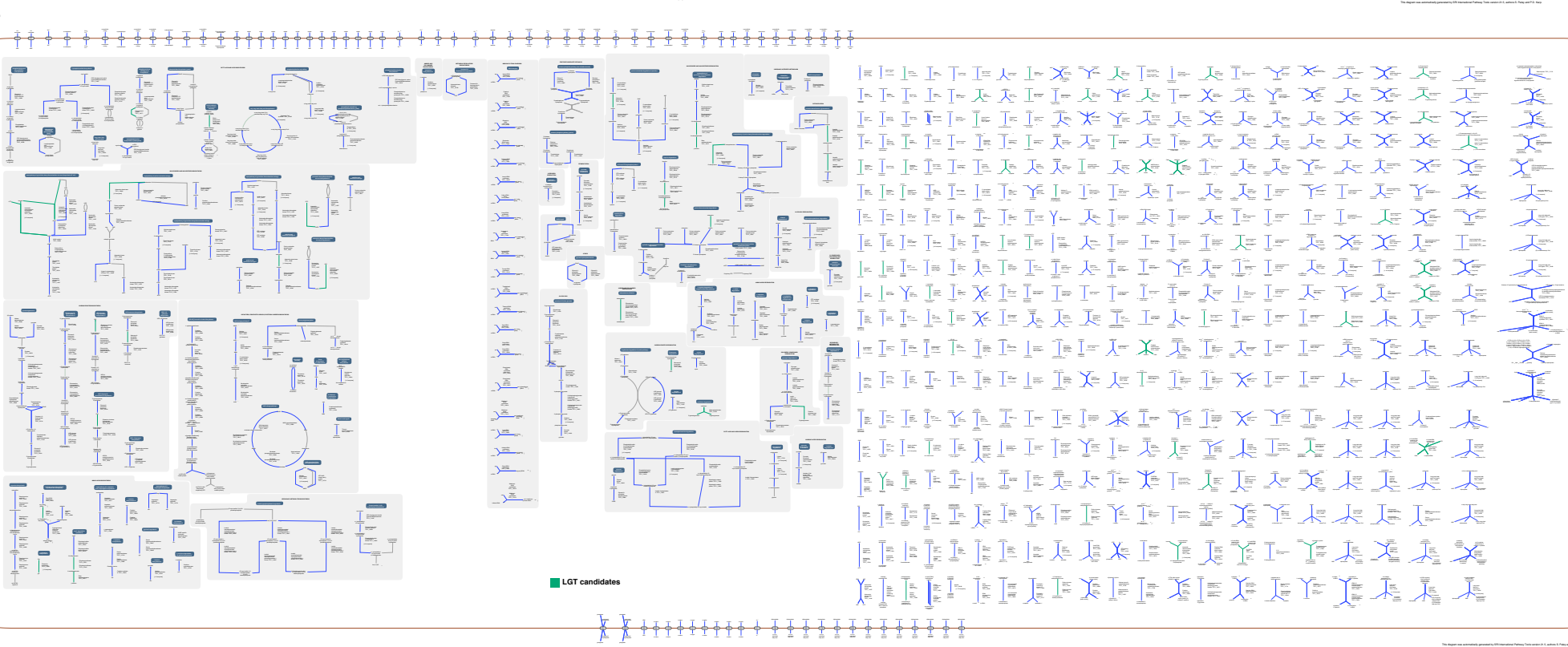

Supplement: FIG S6 [file mSystems.00774-20-sf006.pdf]

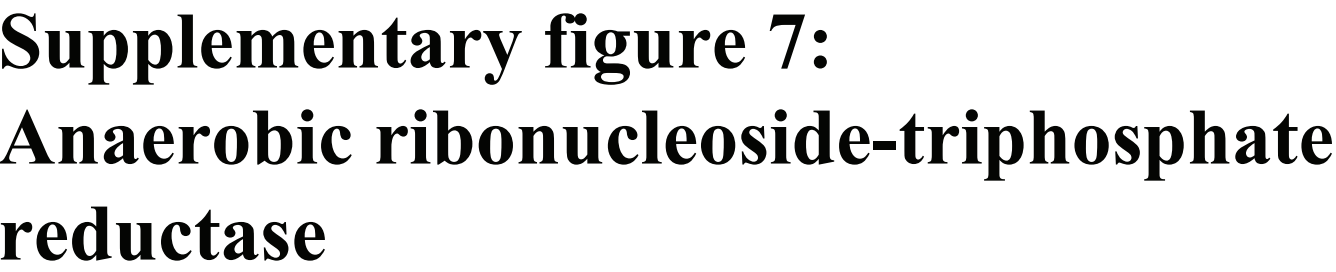

Supplement: FIG S7 [file mSystems.00774-20-sf007.pdf]
