# Supplementary material for: Metabolic Reconstruction Elucidates the Lifestyle of the Last Diplomonadida Common Ancestor
Source: mSystems. 2020 Dec 22;5(6):e00774-20. doi: 10.1128/mSystems.00774-20 (PMC7762791; doi:10.1128/mSystems.00774-20)
Supplement: TABLE S2 [file mSystems.00774-20-st002.pdf]

Supplementary Table 2: Gains and losses in *G. intestinalis* ancestor, *G. intestinalis* WB and *G.intestinalis* GS B.

**LGT candidates**	**Reactions lost**
***G. intestinalis* lineage**	
Flavohemoprotein<br>MsrB	Phosphopantetheine adenyltransferase<br>tRNA(guanine(37)-N1)-methyltransferase<br>Tryptophanase x2<br>Amino acid transporter
***G. intestinalis* WB**	
**LGT candidates**	**Reaction lost**
	2,5-diketo-D-gluconinc acid reductase<br>Quorum-quenching N-acyl-homoserine lactonase<br>Arginase x2<br>Maltose O-acetyltransferase<br>Nicotine N-methyltransferase
***G. intestinalis* GS B**	
**LGT candidates**	**Reaction lost**
Exodeoxyribonuclease VII small subunit<br>L-ascorbate-6-phosphate lactonase	Dolichyl-phosphate-mannose-protein mannosyltransferase<br>Isopentenyl-diphosphote delta-isomerase<br>Ribokinase x2<br>NAD(P)H-hydrate epimerase x2<br>Serine-pyruvate aminotransferase x2<br>Cation efflux family protein
